# Single Nucleotide Polymorphism of Genes Associated with Metabolic Fatty Liver Disease

**DOI:** 10.1155/2022/9282557

**Published:** 2022-02-03

**Authors:** Tong Mu, Linrui Peng, Xinglei Xie, He He, Qing Shao, Xiran Wang, Yuwei Zhang

**Affiliations:** ^1^Department of Endocrinology and Metabolism, West China Hospital, Sichuan University, Chengdu 610041, China; ^2^Department of Clinical Laboratory, West China Hospital, Sichuan University, Chengdu 610041, China; ^3^Department of Preventive Medicine, Sichuan University, Chengdu 610041, China

## Abstract

**Aims:**

The present study aimed to reveal the relationship between single nucleotide polymorphism (SNP) of PNPLA3, TM6SF2, MBOAT7, GATAD2A, and STAT3 genes and metabolism-related fatty liver disease (MAFLD), so as to provide a research basis for further exploring the diagnosis and treatment of diseases at the molecular level.

**Methods:**

A total of 564 patients were included in the physical examination center of Xinjiang Karamay People's Hospital. They were divided into an MAFLD case group and a healthy control group. The whole blood DNA of each sample was extracted by a whole blood genomic DNA extraction kit, and the genotypes of PNPLA3 rs738409, MBOAT7 rs64173, STAT3 rs744166, TM6SF2 rs58542926, and GATAD2A rs4808199 were performed; after adjusting for confounding factors, the additive model, dominant model, and recessive model of each gene were analyzed by multivariate logistic regression.

**Results:**

The CC genotype of the PNPLA3 gene rs738409 and the TT genotype of the MBOAT7 gene rs64173 are risk factors in the occurrence of MAFLD. The AA genotype of the STAT3 gene rs744166 is a protective factor of MAFLD, while TM6SF2 rs58542926 and GATAD2A rs4808199 show no significant correlation with MAFLD.

## 1. Introduction

Metabolism-related fatty liver disease (MAFLD) is a clinicopathological syndrome characterized by diffuse hepatocyte steatosis and lipid storage without excessive drinking history. It may lead to a series of diseases, including steatohepatitis, liver fibrosis, and liver cirrhosis [1], which is a chronic metabolic disease seriously endangering human health. Clinically, the gold standard for the diagnosis of MAFLD is liver biopsy, which has a certain risk. For example, liver puncture may lead to bleeding at the puncture point, bleeding in the abdominal cavity, damage to the surrounding tissues and organs, causing liver injury, bile fistula, intrahepatic arteriovenous fistula, intrahepatic infection, abdominal infection, and puncture to the lungs causing pneumothorax. Therefore, it is particularly important to deeply explore the pathophysiological process of exploring and finding out possible biomarkers for the diagnosis of MAFLD.

MAFLD is a metabolic liver injury closely related to genetic susceptibility, which is related to single nucleotide polymorphisms at multiple related gene loci. Single nucleotide polymorphism (SNP) refers to the DNA sequence polymorphism caused by the change of a single nucleotide at the genomic level, mostly the conversion or transversion of a single base. MALFD is associated with multiple endogenous gene polymorphisms. A current study involving a large sample of European populations is based on genome-wide association analysis (GWAS) and identified five MAFLD susceptibility loci, which are located at or near GCKR, tr1b1, mau2/TM6SF2, ApoE, and PNPLA3, respectively [2]. However, we found that the correlation between TM6SF2 and PNPLA3 gene polymorphisms and MAFLD in the Chinese population is not consistent with that in the European and American populations. In addition to the abovementioned five loci, studies have found MBOAT7, STAT3, and GATAD2A gene polymorphisms may be related to the liver lipid metabolism pathway [3–7]. However, there is no study on the correlation between these gene polymorphisms and MALFD in the Chinese population. Therefore, we selected the gene encoding patatin such as phospholipase domain protein 3 (PNPLA3) rs738409, transmembrane 6 superfamily 2 gene (TM6SF2) rs58542926, membrane bound o-acyltransferase domain 7 gene (MBOAT7) rs64173, signal transduction activating transcription factor-3 gene (STAT3) rs744166, and nuclear small body weight plastic deacetylase gene (GATAD2A) rs4808199, to explore the relationship between these gene loci and MAFLD.

The incidence rate of MAFLD in China's Xinjiang area is much higher than that of Han nationality due to its unique genetic background and special lifestyle [8]. Therefore, we intend to take the Karamay minority population in Xinjiang, China as the research object to explore the relationship between single nucleotide polymorphisms of PNPLA3, TM6SF2, MBOAT7, GATAD2A, and STAT3 genes and metabolic fatty liver, and to explore the mechanism of the abovementioned gene loci affecting the occurrence and development of MAFLD, so as to provide a basis for early diagnosis and individualized early prevention, and a research basis for further exploring the diagnosis and treatment of diseases at the molecular level in the modern medical model.

## 2. Methods

### 2.1. Subjects and Clinical Criteria

564 patients were divided into two groups: the case group and the healthy control group. They are all 28–67 years old and have lived in the Karamay area for more than 10 years. The case group was the patients with MAFLD in Karamay City, Xinjiang, whose weight was stable three months before the start of the study and did not take liver protective drugs and were diagnosed as MAFLD according to imaging examination or pathological biopsy. On the other hand, the control group consisted of normal people without abnormalities in imaging and hematology. The age, sex, nationality, and region of the control group were matched with those of the case group. The exclusion criteria were as follows: history of excessive drinking (alcohol consumption equivalent to more than 30 g/*D* for men and more than 20 g/*D* for women), specific liver diseases (such as genotype 3 HCV infection, autoimmune hepatitis, and hepatolenticular degeneration), drug effects (such as tamoxifen, amiodarone, sodium valproate, methotrexate, and glucocorticoid), total parenteral nutrition, inflammatory bowel disease, celiac disease, hypothyroidism, Cushing's syndrome, *β*-lipoprotein deficiency, lipoatrophy, diabetes, and so on. Next, data were collected, including general information (such as name, nationality, age, gender, height, weight, BMI, smoking history, drinking history, medication history, and past medical history), biochemical indicators (such as fasting blood glucose, triglyceride, cholesterol, high-density lipoprotein, low-density lipoprotein, aspartate aminotransferase, alanine aminotransferase, direct bilirubin, indirect bilirubin, albumin, and uric acid), and liver B-ultrasound results.

The severity of hepatotoxicity was classified according to the WHO toxicity classification standards (Grade 1 (mild), ALT < 2.5 ULN; Grade 2 (mild), ALT 2.5–5 ULN; Grade 3 (moderate), ALT 5–10 ULN; and Grade 4 (severe), ALT > 10 ULN) [9], and different clinical patterns were categorized into hepatocellular, cholestatic, and mixed liver injury based on the *R* value, where *R* = (ALT/ULN)/(ALP/ULN) (ALP, alkaline phosphatase) [10].

#### 2.1.1. Genomic DNA Extraction and Genotyping

A whole blood genomic DNA extraction kit was used to extract whole blood DNA. Primer3 online, Oligo, GeneMapper, SHEsis, and other software were used to design PCR amplification primers and the single-base extension primers for the polymorphic sites to be tested. Multiplex PCR technology and single-base extension technology were combined for genotyping detection.

#### 2.1.2. Statistical Analysis

Unless stated otherwise, all statistical analyses were performed with SPSS 20.0 (SPSS, Munich, Germany) or GraphPad Prism 5.0 (GraphPad Software Inc., CA, USA). Quantitative data were expressed as medians and ranges. Exact tests were performed to check the consistency of genotyping results with Hardy–Weinberg equilibrium (HWE). Under the additive, dominant, and recessive genetic models, linear regression was used to analyze the correlation between each gene locus and MAFLD. All models were adjusted for confounding factors.

## 3. Results

### 3.1. Demographical and Clinical Data

Among the 282 ATLI cases, a total of 110 (39.0%) ATLI cases had Grade 1 (mild) hepatotoxicity, 107 (37.9%) had Grade 2 (mild) hepatotoxicity, 46 (16.3%) had Grade 3 (moderate) hepatotoxicity, and 19 (6.8%) had Grade 4 (severe) hepatotoxicity. There were no significant differences in the baseline characteristics of the two groups except for weight, BMI, and triglycerides (Table 1).

### 3.2. Genotype Analysis

Hardy–Weinberg equilibrium tests demonstrated no significant deviation from the expected values for the five tagSNPs among the controls (rs738409, *χ*^2^ = 1.137, *P*=0.478; rs641738, *χ*^2^ = 0.340, *P*=0.286; rsrs744166, *χ*^2^ = 0.938, *P*=0.130; rs58542926, *χ*^2^ = 0.503, *P*=0.560; and rsrs4808199, *χ*^2^ = 0.013, *P*=0.338) (Table 2).

### 3.3. Gene Single Nucleotide Polymorphism and MAFLD

After correcting for potential confounding factors, conditional logistic regression analysis (Table 3) showed that patients carrying the CC genotype at rs738409 in PNPLA3 were at a higher risk of MAFLD than those with the GG and GC genotypes (adjusted OR = 1.402, 95% CI: 1.026–2.239, *P*=0.033), and significant differences were also found under the recessive (*P*=0.016) and additive models (*P*=0.046). The TT genotype of MBOAT7 rs64173 was a risk factor in the occurrence of MAFLD (*P*=0.02), while the AA genotype of STAT3 rs744166 can serve as a protective factor in the occurrence of MAFLD (*P*=0.016). TM6SF2 and GATAD2 had no significant correlation with the occurrence of MAFLD. However, no other significant differences in the genotypes of the remaining genes were observed between patients and healthy controls.

### 3.4. Subgroup Analysis

According to the extended subgroup analysis of liver injury severity, risk related tagSNP-PNPLA3 rs738409 was still a risk factor in Grade 1 and Grade 2 (mild) cases under implicit and additive models (or = 1.614, *P*=0.009; or = 1.279, *P*=0.038), but there was no correlation in Grade 3 and Grade 4 (moderate and severe) cases (*P* > 0.05) (Table 4).

## 4. Discussion

As a common chronic disease, the pathogenesis of metabolism-related fatty liver disease has not been clearly determined. Current studies mostly believe that it is the result of multiple factors such as polygenic genetic variation and the environment. In a European population study of 423252 people, the researchers constructed a polygenic risk score (GRS) related to metabolism-related fatty liver disease. It was found that a higher GRS can significantly amplify the risk of MAFLD for liver-related diseases, and the risk is further amplified with age [11]. This suggests that there is a close correlation between metabolism-related fatty liver disease and endogenous genes.

With the upgrading of gene screening methods and the development of human genome projects, whole genome scanning (GWAS) is widely used in the related gene screening of MAFLD, promoting the diagnosis of diseases, the development of new drugs, and the exploration of new therapies. Among them, single nucleotide polymorphism, that is, the study of single nucleotide substitution in the genome, plays an increasingly important role in gene mapping and association analysis of complex diseases. The latest genome-wide analysis results show that in 770180 case-control studies, researchers have identified five MAFLD susceptibility loci through genome-wide meta-analysis, which are located in GCKR, tr1b1, mau2/TM6SF2, and ApoE, respectively, and PNPLA3 [2]. However, the correlation between TM6SF2 and PNPLA3 gene polymorphisms and MAFLD in the Chinese population is different from that in the European population. According to a study of 1200 Chinese people, the CC genotype of PNPLA3 rs738409 is a risk factor for MAFLD (*P*=0.046) [12], while the GC genotype is more prone to MAFLD than the CC genotype in the European population [3, 13]. We know that most of the more than 100 loci identified by GWAS are found in the European population, while the weight of different loci is different in various ethnic groups [14]. Therefore, the correlation between TM6SF2 and PNPLA3 gene polymorphisms and MAFLD still needs to be improved in the Chinese population with the help of a lot of evidence-based medical evidence.

In our study, taking fatty liver as the dependent variable and adjusting body weight, BMI, and other factors, multivariate logistic analysis was carried out on the additive model, dominant model, and recessive model of each gene. The CC gene of PNPLA3 is a risk factor in the occurrence of MAFLD, and the TT gene of MBOAT7 carries out the same job. The AA gene of STAT3 is a risk factor in the occurrence of MAFLD, while TM6SF2 and GATAD2A have no significant correlation with the occurrence of fatty liver.

Based on the abovementioned results, our study shows that PNPLA3, MBOAT7, and STAT3 affect the occurrence and development of MAFLD. The PNPLA3 gene mutation is associated with increased transaminase activity, which can promote the progression of liver fibrosis and steatosis [15–17]. MBOAT7 can catalyze the desaturation of the second acyl chain of phospholipids and transfer polyunsaturated fatty acids [18]. STAT3 plays a key role in the process of leptin resistance, and high leptin levels may be related to the increase of nonalcoholic fatty liver disease [19]. In addition, STAT3 is downregulated in atherosclerotic lesions of ApoE -/- mice. Its forced overexpression can reduce inflammation, lipid accumulation, and vascular smooth muscle cell proliferation, indicating that it has atherosclerotic protective function [20]. TM6SF2 and GATAD2A are also related to MAFLD in foreign studies [21, 22], but they are not related in our study of Xinjiang ethnic minorities.

The PNPLA3 gene is a member of the non-Ca2 + − dependent patatin like phospholipase family on chromosome 22 [23]. The protein encoded by the PNPLA3 gene is a nonsecretory protein adiponutrin composed of 481 amino acids. It is a four times transmembrane protein, which mainly exists on the cell membrane and cytoplasmic lipid droplets [24]. PNPLA3 is a genetic variation highly related to the occurrence and development of MAFLD. Studies have shown that GG homozygous variants in European populations increase the risk of HCC associated with MAFLD by 10 times [25]. Interestingly, PNPLA3 carriers have a significantly increased risk of cirrhosis and hepatocellular carcinoma, independent of the tendency to steatosis. This indicates that PNPLA3 is directly involved in the process of fiber formation and carcinogenesis [26].

In 2015, MBOAT7rs641738 was first found to be closely associated with metabolic cirrhosis in a European study including 1148 patients with metabolic cirrhosis and 922 healthy controls. Researchers from the European Dallas Research Center genotyped MBOAT7rs641738 in 1149 patients who underwent liver biopsy. The results showed that the MBOAT7rs641738 polymorphism was associated with liver fat content, and patients with *T* allele were at greater risk of serious liver injury and fibrosis. In individuals with a site mutation, the expression of MBOAT7 was downregulated at mRNA and protein levels, and the levels of phosphatidylinositol in hepatocytes and circulating blood were also changed [27]. Researchers from Italy found that carrying the MBOAT7 T allele is an independent risk factor for MAFLD related liver cancer [28]. Contrary to the abovementioned population studies, the study from Taiwan children in China detected the MBOAT7rs641738 gene polymorphism in 831 obese MAFLD children aged 7∼15. The results showed that the MBOAT7rs641738 gene polymorphism was associated with steatohepatitis, insulin resistance, and blood lipid levels. There was no significant correlation between liver enzyme levels and serum CK18 levels [29]. The results of this study showed that TT genotype at the MBOAT7rs641738 locus increased the risk of MAFLD, which was consistent with the data of the European population.

In our study, STAT3 gene polymorphism was associated with MAFLD. The activation of STAT3 eventually leads to cytokine signal inhibitor-3 (SOCS-3) playing a feedback inhibitory role by weakening the OBRb signal and plays a key role in leptin resistance in part by binding tyr985 [30]. Leptin is considered to be an antitear hormone, which can protect nonadipose tissues including the liver, from fat accumulation and fat toxicity. However, in the state of insulin resistance, including obesity, it is not only high and thin. The protective effect of leptinemia seems to be limited, and hyperleptinemia may have adverse effects, thus promoting insulin resistance, hepatic steatosis, inflammation, fibrosis, and carcinogenesis.

In summary, MAFLD is a complex disease related to genetic, environmental, and metabolic stress. Its pathogenesis is affected by many factors, such as genetics, environment, immunity, nutrition, and so on [31, 32]. By affecting lipid metabolism, inflammatory response, insulin resistance, oxidative stress, liver fibrosis and other processes, gene polymorphism, and epigenetics can regulate the susceptibility and progression of MAFLD [33]. Gene polymorphism provides ideas for the early diagnosis and targeted therapy of MAFLD and provides a theoretical basis for the creation and secondary development of new drugs.

## Figures and Tables

**Table 1:** Characteristics of patients in MAFLD cases and controls.

Characteristic	MAFLD cases (*n* = 282)	Controls (*n* = 282)	*P* value

Gender (male/female)	145/137	142/140	—
Age (y)	47.8 ± 19.1	47.7 ± 19.0	0.868
Weight (kg)	57.3 ± 10.6	55.8 ± 10.0	0.004
BMI (kg/m^2^)	24.83 (18.61–30.47)	20.12 (18.51–23.80)	0.018
Glycosylated hemoglobin (%)	5.6 (4.6–8.1)	5.3 (4.0–6.0)	0.067
ALT (U/L)	19.0 (7.0–79.0)	16.0 (5.0–31.0)	0.090
AST (U/L)	22.0 (10.0–82.0)	20.0 (17.0–27.0)	0.053
Triglycerides (mmol/L)	1.79 (0.66–5.38)	1.09 (0.38–1.60)	0.048
Cholesterol (mmol/L)	3.31 (1.69–8.33)	2.82 (0.87–4.78)	0.749
Low-density lipoprotein (mmol/L)	2.96 (0.99–7.17)	2.51 (0.19–3.19)	0.690

**Table 2:** Information on five tagSNPs.

Gene	tagSNPs	Chromosome position	Base change	HWE *P* value

PNPLA3	rs738409	35777618	*G* > *C*	0.478
MBOAT7	rs641738	45782513	*C* > *T*	0.286
STAT3	rs744166	11307603	*G* > *A*	0.130
TM6SF2	rs58542926	55786873	*C* > *T*	0.560
GATAD2A	rs4808199	69743760	*G* > *A*	0.338

**Table 3:** Genotypes distribution in two groups and the risks of MAFLD.

Gene	tagSNPs	Cases (*n* = 282)		Controls (*n* = 282)		OR (95% CI)	*P* value	Model	OR (95% CI)	*P* value
		*n*	%	*n*	%					

PNPLA3	rs738409 (*G* > *C*)									
	GG	56	19.9	68	24.1	1.000		Dom	1.160 (0.824–1.615)	0.424
	GC	152	53.9	161	57.1	1.006 (0.713–1.447)	0.859	Rec	1.492 (1.080–2.066)	0.016
	CC	74	26.2	53	18.8	1.402 (1.026–2.239)	0.033	Add	1.232 (1.003–1.504)	0.046
MBOAT7	rs64173 (*C* > *T*)									
	CC	53	18.8	64	22.7	1.000		Dom	1.018 (0.778–1.459)	0.848
	CT	159	56.4	167	59.2	1.003 (0.742–1.359)	0.979	Rec	1.398 (1.053–1.692)	0.032
	TT	70	24.8	51	18.1	1.299 (0.933–1.748)	0.040	Add	1.038 (0.856–1.259)	0.061
STAT3	rs744166 (*G* > *A*)									
	GG	55	19.5	69	24.5	1.000		Dom	1.193 (0.957–1.491)	0.398
	GA	150	53.2	159	56.4	1.223 (0.842–1.573)	0.947	Rec	0.682 (0.372–1.195)	0.019
	AA	77	27.3	54	19.1	0.738 (0.469–1.201)	0.026	Add	1.004 (0.894–1.356)	0.059
TM6SF2	rs58542926 (*C* > *T*)									
	CC	60	21.3	71	25.2	1.000		Dom	0.973 (0.765–1.320)	0.883
	CT	155	54.9	146	51.8	1.005 (0.782–1.392)	0.969	Rec	0.915 (0.657–1.280)	0.615
	TT	67	23.8	65	23.0	0.928 (0.631–1.336)	0.655	Add	0.952 (0.792–1.169)	0.699
GATAD2A	rs4808199 (*G* > *A*)									
	AA	54	19.1	42	14.9	1.000		Dom	1.042 (0.769–1.415)	0.789
	AG	164	58.2	162	57.4	0.980 (0.719–1.364)	0.957	Rec	1.687 (0.734–3.897)	0.217
	GG	64	22.7	78	27.7	1.678 (0.719–3.883)	0.218	Add	1.088 (0.836–1.416)	0.536

**Table 4:** Subgroup analysis among different severity of hepatotoxicity with different genetic models.

Genes	tagSNPs	Model	Grade 4 and 3 (*n* = 65)	*P* value	Grade 2 and 1 (*n* = 217)	*P* value
			OR (95% CI)		OR (95% CI)	

PNPLA3	rs738409 (*G* > *C*)					
	GG	Dom	1.198 (0.482–2.479)	0.683	1.121 (0.685–1.633)	0.507
	GC	Rec	1.052 (0.581–1.876)	0.749	1.614 (1.156–2.506)	0.009
	CC	Add	1.079 (0.754–1.732)	0.738	1.279 (1.010–1.628)	0.038
MBOAT7	rs64173 (*C* > *T*)					
	CC	Dom	0.796( 0.373–1.919)	0.683	0.787 (0.463–1.349)	0.298
	CT	Rec	–	–	2.013 (0.115–5.97)	0.654
	TT	Add	0.861 (0.392–1.896)	0.687	0.830 (0.519–1.316)	0.438
STAT3	rs744166 (*G* > *A*)					
	GG	Dom	1.417 (0.812–2.389)	0.221	1.219 (0.891–1.672)	0.229
	GA	Rec	0.827 (0.355–1.863)	0.675	1.464 (0.862–2.483)	0.150
	AA	Add	1.152 (0.891–1.810)	0.488	1.217 (0.961–1.533)	0.135
TM6SF2	rs58542926 (*C* > *T*)					
	CC	Dom	1.011 (0.524–1.838)	0.921	0.885 (0.629–1.252)	0.518
	CT	Rec	0.787 (0.376–1.632)	0.528	1.062 (0.729–1.537)	0.886
	TT	Add	0.954 (0.662–1.399)	0.792	0.973 (0.772–1.219)	0.769
GATAD2A	rs4808199 (*G* > *A*)					
	AA	Dom	0.618 (0.345–1.096)	0.095	1.215 (0.885–1.669)	0.266
	AG	Rec	1.122 (0.519–2.481)	0.789	1.090 (0.712–1.683)	0.693
	GG	Add	0.816 (0.540–1.226)	0.316	1.125 (0.896–1.387)	0.392

## Data Availability

The datasets used and/or analyzed during the current study are available from the corresponding author on reasonable request.
